# Curcumin Versus Corticosteroids for Symptomatic Oral Lichen Planus: A Systematic Review and Meta‐Analysis

**DOI:** 10.1002/cre2.70227

**Published:** 2025-09-22

**Authors:** Sadeq A. Al‐Maweri, Gamilah Al‐Qadhi, Ahmed Yaseen Alqutaibi, Nadhem M. Sallam, Mounzer Assad, Mahfoudh A. Abdulghani, Marwan Mansoor Ali Mohammed

**Affiliations:** ^1^ College of Dental Medicine, QU Health Qatar University Doha Qatar; ^2^ Department of Basic Dental Sciences, Faculty of Dentistry University of Science and Technology Aden Yemen; ^3^ Department of Prosthodontics, College of Dentistry Taibah University Medina Saudi Arabia; ^4^ Department of Paediatric and Preventive Dentistry, Faculty of Dentistry Thamar University Dhamar Yemen; ^5^ Department of Oral Surgery, Faculty of Dentistry Tishreen University Lattakia Syria; ^6^ Pharmacology Department, International Medical School Management and Science University Shah Alam Selangore Malaysia; ^7^ Department of Oral and Craniofacial Health Sciences, College of Dental Medicine University of Sharjah Sharjah United Arab Emirates

**Keywords:** corticosteroids, curcumin, efficacy, oral lichen planus, systematic review

## Abstract

**Objectives:**

The management of oral lichen planus (OLP) is challenging, with no definitive cure available. This systematic review and meta‐analysis aimed to assess the efficacy of curcumin in managing OLP.

**Material and Methods:**

A comprehensive search of PubMed, Scopus, Web of Science, and Google Scholar was conducted for relevant clinical trials published up to March 31, 2025. All clinical trials comparing the efficacy of curcumin to corticosteroids were included. A meta‐analysis was conducted on studies with available numerical data to assess the efficacy of curcumin relative to the control group.

**Results:**

Eleven clinical trials, involving 499 OLP patients, were included in this review, with nine studies were incorporated into the meta‐analysis. All studies reported curcumin to be efficacious in alleviating the signs and symptoms of OLP. Pooled data showed significantly better efficacy of curcumin in reducing pain at 1 week (SMD, −0.70; 95% CI, −1.33, −0.07; *p* = 0.03; *I* ^2^ = 66%) compared with corticosteroids. However, no significant differences were observed between the two groups at the 2‐, 4‐, and 12‐week follow‐ups. Additionally, curcumin and corticosteroids demonstrated comparable efficacy in clinical improvement of lesions across different time points.

**Conclusions:**

The available evidence suggests that curcumin has promising effects in managing OLP. However, due to methodological limitations, including significant heterogeneity among the studies and a high risk of bias in most of them, further well‐designed studies with adequate follow‐up periods are needed.

## 1 Introduction

Oral lichen planus (OLP) is a chronic inflammatory disorder of the oral mucosa, affecting 0.47%–1.74% of the general population (González‐Moles et al. 2021). OLP can present in different clinical forms, including reticular, papular, plaque‐type, erosive, and atrophic (Carrozzo et al. 2019; Vičić et al. 2023). While the hyperkeratotic types (reticular, papular, plaque‐type) are mostly asymptomatic, the atrophic‐erosive type is mostly associated with severe pain and discomfort that interferes with daily functions and adversely affects patients' quality of life (Daume et al. 2021). Additionally, OLP has recently been classified as oral potentially malignant disorder, with an estimated transformation rate of 1.14% (95% CI = 0.84–1.49) (González‐Moles et al. 2019; Chu et al. 2025). Although the exact etiopathogenesis of OLP is still unclear, mounting evidence indicates an immunological response to unclear triggers (Vičić et al. 2023; DeAngelis et al. 2019; Maneerat et al. 2025).

Despite the extensive research on the topic, the management of OLP is still quite challenging with no definitive cure (Yang et al. 2016; Kiyani et al. 2021; Chirravur et al. 2024). Various pharmacological (such as corticosteroids, tacrolimus, herbal agents, and immunosuppressants) and nonpharmacological (such as laser therapy and photodynamic therapy) modalities have been used for the management of OLP with limited success (Yang et al. 2016; Chirravur et al. 2024; García‐Pola et al. 2017; Lodi et al. 2020; Su et al. 2022; Al‐Maweri, Ashraf, et al. 2018; Al‐Maweri et al. 2017; Liu et al. 2025). Among these, topical and systemic corticosteroids have been the first‐line therapy for OLP treatment (Lodi et al. 2020). However, corticosteroids are often associated with several oral and systemic complications that limit their use (Khater and Khattab 2020; Al‐Maweri et al. 2023). Hence, investigators have been in search of new alternative therapeutic agents for the management of OLP.

The use of herbal products for the management of oral diseases has gained a lot of interest in recent years. Among these, curcumin (also known as turmeric) has been of great interest for the management of oral inflammatory diseases (Tang et al. 2021). Curcumin is a herbal product extracted from the roots of *Curcuma longa* (Sohn et al. 2021; Witkin and Li 2013). It has been used in medicine for the treatment of several medical conditions, including but not limited to rheumatoid arthritis, inflammatory bowel diseases, and dermatological conditions (Ebrahimzadeh et al. 2021; Barbalho et al. 2021). The medicinal benefits of curcumin are ascribed to its potent analgesic, anti‐inflammatory, antioxidant, and immunomodulatory properties (Witkin and Li 2013; Ferguson et al. 2021). Additionally, curcumin has been shown to have anticancer properties (Ohnishi et al. 2020). Such properties make curcumin a good candidate for the management of oral inflammatory disorders, including OLP. In this context, a number of clinical trials have assessed the efficacy of curcumin in the management of OLP, and revealed very promising results (Kia et al. 2020, 2015; Nosratzehi et al. 2018; Tantia and Gupta 2021; Thomas et al. 2017). Although some reviews have highlighted the efficacy of curcumin in the management of oral diseases, including OLP (Lv et al. 2019; Dharman and Ravinthar 2020; Tang et al. 2021; Moayeri et al. 2024), no attempt has been made so far to quantitatively analyze the available evidence on the efficacy of curcumin for OLP in comparison to corticosteroids, the mainstay therapy for OLP. Hence, the present systematic review and meta‐analysis aimed to summarize and appraise the available evidence regarding the efficacy of curcumin in reducing signs and symptoms of OLP. The formulated Participants, Intervention Control, and Outcome (PICO) question was “Compared to corticosteroids (C), what is the efficacy of curcumin (I) in reducing signs and symptoms (O) of patients with OLP (P)?”

## 2 Methods

The present systematic review and meta‐analysis were conducted in full compliance with the PRISMA 2020 guidelines and PICO principles (Page et al. 2021).

### 2.1 Eligibility Criteria

The PICO‐based eligibility criteria were as follows:

P (Population): Patients ≥ 15 years diagnosed with OLP.

I (Intervention): Curcumin agents (topically or systemically).

C (Control): Corticosteroids.

O (Outcome): The primary outcomes were subjective (pain/burning sensation) and objective improvement (changes in the erosion/erythema size). The secondary outcomes: side effects of the interventions.

S (Study design): All randomized clinical trials (RCTs) and nonrandomized clinical trials (NRCTs).

Case series, reviews, animal studies, uncontrolled trials, and studies with a small sample size (10 in each group) were excluded.

### 2.2 Search Strategy Information Sources

Two investigators (S.A. and G.A.) independently conducted a comprehensive literature search in PubMed, Scopus, Web of Science, and Google Scholar on June 16, 2022. The search was updated on April 4, 2025, to include all relevant studies published from the inception until March 31, 2025. The following Mesh terms and free keywords were used: (“Lichen Planus, Oral” [Mesh] OR “Oral lichen planus”) AND “Curcumin” [Mesh] OR curcumin OR turmeric). The gray literature was searched via ProQuest (Table S1 presents details of the search strategy). In addition to the online searches, a manual search of the retrieved references was conducted for any additional studies. Authors of respective articles were contacted for any missing data or clarification. There was no restriction on the date or language.

### 2.3 Study Selection

The identified studies were exported to an EndNote program Version X9, and duplicate records were removed. The two reviewers (S.A. and G.A.) independently screened the titles and abstracts of the identified articles, and irrelevant studies were eliminated. The full‐text of all potentially eligible studies was then sought and assessed by the two reviewers for inclusion.

### 2.4 Quality Assessment

The quality of each included study was appraised by two investigators (S.A. and G.A.) independently, using the revised Cochrane risk‐of‐bias tool for randomized trials (Sterne et al. 2019). Five domains were evaluated: randomization process, deviations from intended interventions, missing outcome data, measurement of the outcome, and selection of the reported result. Accordingly, the overall risk of bias (ROB) for each study was judged as either “low,” “high,” or “some concerns” (Sterne et al. 2019).

### 2.5 Data Extraction

Two investigators (S.A. and G.A.) independently extracted and tabulated the relevant data using special data collection forms including the following: author's name and country of publication, study design, dose and frequency of the intervention, dose and frequency of the controls, type of OLP, sample size, participants' age and gender, follow‐up period, outcome measures, and the main outcomes.

### 2.6 Meta‐Analysis

Review Manager (RevMan) Version 5.3 was used for data analysis. The meta‐analyses were conducted by calculating the standardized mean difference (SMD) in the pain and clinical improvement scores between the two groups, along with 95% confidence intervals (CIs). Heterogeneity between the studies was evaluated using the Chi‐square test and the *I* ^2^ statistic. The potential publication bias was assessed using the funnel plots. Due to the limited number of the included studies, no sensitivity tests or subgroup analyses were conducted.

## 3 Results

### 3.1 Study Selection

**Figure 1:**
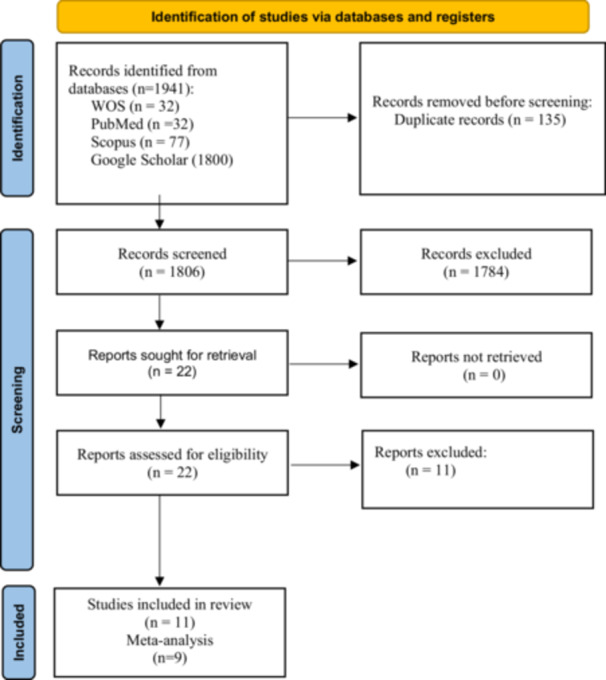
Flow diagram of the search strategy. WOS, Web of Science.

The online searches yielded 1941 records, of which 135 were duplicates and thus excluded (Figure 1). The titles and abstracts of the remaining 1806 articles were screened for eligibility, and 1784 were excluded for being irrelevant. The full‐text of the potentially eligible 22 articles was carefully read for eligibility, and 11 were excluded for various reasons (Table S2). Eventually, 11 studies were included in the present systematic review (Figure 1).

### 3.2 General Characteristics of the Included Studies

The characteristics of the included studies are illustrated in Table 1. A total of 11 clinical trials were included in the present systematic review (Kia et al. 2020, 2015; Nosratzehi et al. 2018; Tantia and Gupta 2021; Thomas et al. 2017; Biswas et al. 2018; Keshari et al. 2015; Pai and Ganesan 2018; Patel et al. 2022; Singh et al. 2021; Kotb et al. 2025), nine of which were included in the meta‐analysis (Kia et al. 2020, 2015; Nosratzehi et al. 2018; Thomas et al. 2017; Biswas et al. 2018; Keshari et al. 2015; Pai and Ganesan 2018; Patel et al. 2022; Kotb et al. 2025). Ten of the included studies were RCT (Kia et al. 2020, 2015; Tantia and Gupta 2021; Thomas et al. 2017; Biswas et al. 2018; Keshari et al. 2015; Pai and Ganesan 2018; Patel et al. 2022; Singh et al. 2021; Kotb et al. 2025), and one was NRCT (Nosratzehi et al. 2018). Seven studies were conducted in India (Tantia and Gupta 2021; Thomas et al. 2017; Biswas et al. 2018; Keshari et al. 2015; Pai and Ganesan 2018; Patel et al. 2022; Singh et al. 2021), three in Iran (Kia et al. 2020, 2015; Nosratzehi et al. 2018), and one in Egypt (Kotb et al. 2025). The studies were published between 2015 and 2024, and involved 499 OLP patients (263 in the curcumin groups and 236 in the corticosteroids groups), with ages ranging from 20 to 78 years. Female subjects comprised the majority of the study participants (over 70%). Six studies confirmed the diagnosis of OLP histopathologically, while five studies (Biswas et al. 2018; Keshari et al. 2015; Pai and Ganesan 2018; Patel et al. 2022; Kotb et al. 2025) relied on clinical features for diagnosis (Table 1).

**Table 1:** General characteristics of the included studies.

Author and country	Study design	Type of OLP and location	Curcumin group formulation sample size M/F Age mean/range	Corticosteroid group formulation sample size M/F Age mean/range	Outcomes assessed	Duration follow‐up	Side effects	Main results
Keshari et al. (2015)/India	RCT	Atrophic/erosive NA	Curcumin oral paste three times daily for 2 weeks (*n* = 15) 8/7 Mean age = 44.46 Age range = (31–60)	0.1% TA three times daily for 2 weeks (*n* = 12) 8/4 Mean age = 45.92 Age range = (28–78)	Pain (NRS) Clinically (MOMI)	2 W Patients were evaluated every week for 2 weeks	Curcumin: None Control: Candidiasis *n* = 3	Curcumin was slightly more efficacious than corticosteroid in reducing pain and ulceration areas without significant differences between the groups. However, there was a significant improvement in erythema scores in curcumin group compared with corticosteroid (*p* = 0.002).
Kia et al. (2015)/Iran	RCT	Atrophic, ulcerative B (most) followed by G, T, P, L, F	5% curcumin oral paste three times daily for 4 weeks (*n* = 25) 10/15 Mean age = 49.24 Age range = (38–73)	0.1% TA oral paste three times daily for 4 weeks (*n* = 25) 4/21 Mean age = 52.8 Age range = (38–73)	Pain (VAS) Clinical: (TSS)	4 W Patients were evaluated at the end of the 2nd and 4th weeks	Curcumin: BS ∗ *n* = 7 itching, mild swelling, xerostomia ∗ *n* = NA Control:: BS ∗ *n* = 1 desquamation *n* = 1	Curcumin was as efficacious as corticosteroid in reducing pain, and clinical signs‐associated OLP without any significant differences between the two groups.
Thomas et al. (2017)/India	RCT	Erosive NA	GI: 1% curcumin oral gel three times daily for 3 months (*n* = 25) 19/56 GII: 1% curcumin oral gel six times daily for 3 months (*n* = 25) 19/56 Mean age = 47.7 Range = (20–70)	0.1% TA oral paste three times daily for 3 months (*n* = 25) 19/56 Mean age = 47.7 Age range = (20–70)	BS (NRS) Clinically (MOMI)	12 W Patients were evaluated every 2 weeks for 3 months	Curcumin: None Control: NA	Curcumin (2 regimes) and corticosteroid showed significant improvement in all parameters (*p* < 0.001). However, the maximum reduction in BS, E, and U scores was observed in corticosteroid group.
Biswas et al. (2018)/India	RCT	NA NA	1% curcumin oral paste two times daily for 2 months (*n* = 30) 3/27 Age range = (26–61)	0.1% TA oral paste two times daily for 2 months (*n* = 30) 8/22 Age range = (24–65)	BS (VAS) Clinically (TSS)	8 W evaluated every 2 weeks for 2 months, and followed up every month for 6 months	Curcumin: None Control: Candidiasis *n* = 6	Curcumin was as efficacious as corticosteroid in reducing burning sensation, and clinical signs‐associated OLP without any significant differences between the two groups.
Nosratzehi et al. (2018)/Iran	NRCT	Atrophic/erosive B (most) followed by G, T	Curcumin oral paste three times daily for 3 months (*n* = 20) 9/11 Mean age = 41.9 Age range = (28–60)	0.1% Betamethasone lotion three times daily with nystatin suspension for 3 months (*n* = 20) 5/15 Mean age = 38.5 Age range = (28–60)	BS/Pain (VAS) Clinically (TSS)	12 W Patients were evaluated every week for 2 weeks, then at the end of 1st, 2nd, and 3rd months, and followed up at the end of 1 year	Curcumin: None Control: None	Curcumin was as efficacious as corticosteroid in reducing pain, lesion size, and clinical signs‐associated OLP without any significant differences between the two groups.
Pai and Ganesan (2018)/India	RCT	Atrophic/erosive NA	Curcumin oral paste three times daily for 2 weeks (*n* = 10) 4/6 Mean age = 42.1 range = (21–70)	0.1% TA oral paste three times daily for 2 weeks (*n* = 10) 5/5 Mean age = 45.7 range = (32–60)	BS (NRS) Clinically (MOMI)	2 W Patients were evaluated every week for 2 weeks	Curcumin: None Control: None	Both groups showed improvement in pain and clinical signs, but curcumin exhibited a significant better reduction in BS (*p* = 0.001), and erythema scores (*p* = 0.02) compared with corticosteroid group.
Kia et al. (2020)/Iran	RCT	Atrophic/erosive NA	80 mg Nanocurcumin soft gel capsule once daily for 4 weeks (*n* = 29) 4/25 Mean age = 51.86	10 mg Prednisone capsule once daily for 4 weeks, then tapering to 5 mg within 10 days (*n* = 28) 5/23 Mean age = 53.67	Pain (VAS) Clinically (TSS)	4 W Patients were evaluated every week for 2 weeks, and at the end of the 4th week	Noun	Curcumin was as efficacious as prednisone in reducing the levels of pain, BS, and lesion size without any significant differences between the two groups.
Singh et al. (2021)/India	RCT	Atrophic/erosive B (most) followed by T, L, G	Curcumin oral paste three times daily for 2 weeks (*n* = 25) 11/14 Mean age = 55	0.1% TA oral paste three times daily for 2 weeks (*n* = 25) 12/13 Mean age = 53	BS/Pain (VAS) Clinically (TSS)	2 W Patients were evaluated every week for 2 weeks	Curcumin: None Control: None	A marked improvement in erythema scores in curcumin group compared with corticosteroid group. However, both groups showed similar reduction in pain and ulceration areas without significant differences between the two groups.
Tantia and Gupta (2021)/India	RCT	Atrophic/ulcerative B (most) followed by G, L, T, F, P	5% Curcumin oral paste three times daily for 4 weeks (*n* = 32) 11/21 Mean age = 43.7	0.1% TA oral paste three times daily for 4 weeks (*n* = 32) 10/22 Mean age = 45.2	BS/Pain (VAS) Clinically (TSS)	4 W Patients were evaluated at the end of 2nd and 4th weeks	NA	Curcumin showed slightly better outcomes than corticosteroid in reducing pain and clinical signs‐associated OLP. But no significant differences between the two groups.
Patel et al. (2022)/India	RCT	Reticular, atrophic, erosive NA	1% Curcumin oral paste three times daily for 3 months (*n* = 17) 10/26 Age range = (30–70)	0.1% TA oral paste three times daily for 3 months (*n* = 19) 10/26 Age range = (30–70)	BS/Pain (VAS) Clinically (TSS)	12 W evaluated every week for 2 weeks, then at the end of 1st and 3rd months	Curcumin: None Control: Candidiasis *n* = 1	Curcumin was as efficacious as corticosteroids in reducing the levels of pain and lesion size without any differences between the two groups.
Kotb et al. (2025)	RCT	Reticular, atrophic, erosive NA	1% Curcumin gel six times daily for 6 weeks (*n* = 10) 95% Females 48.44 ± 11.40	0.1% TA oral paste four times per day for 6 weeks (*n* = 10) 95% Females 48.44 ± 11.40	‒ BS/pain (VAS) ‒ Severity score ‒ Site score ‒ IL‐6	4 and 12 weeks	NA	Both groups showed significant decrease in signs and symptoms of OLP, with slight better results in favor of TA group. Additionally combination of TA and curcumin revealed better results than TA or curcumin alone.

Abbreviations: B, buccal mucosa; BS, burning sensation; E, erythema; F, floor of mouth; G, gingiva; IL‐6, Interleukin‐6; L, labial mucosa; MOMI, Maternal Omics to Maximize Immunity; MOMI, modified oral mucositis index; NA, not available; NRCT, nonrandomized clinical trial; NRS, Numerical Rating Scale; OLP, oral lichen planus; P, palate; RCT, randomized clinical trial; T, tongue; TA, triamcinolone acetonide; TSS, Thongprasom Sign Scores; U, ulceration; VAS, visual analog scale.

With respect to the type of OLP cases, eight studies (Kia et al. 2020, 2015; Nosratzehi et al. 2018; Tantia and Gupta 2021; Thomas et al. 2017; Keshari et al. 2015; Pai and Ganesan 2018; Singh et al. 2021) included atrophic‐erosive OLP, two studies (Patel et al. 2022; Kotb et al. 2025) included both reticular and atrophic‐erosive, while one study (Biswas et al. 2018) did not mention the type of OLP. The reported duration of the OLP therapy in the included studies ranged from 2 to 12 weeks (Table 1).

### 3.3 Formulation and Dose of Interventions

Ten studies used topical curcumin (Kia et al. 2015; Nosratzehi et al. 2018; Tantia and Gupta 2021; Thomas et al. 2017; Biswas et al. 2018; Keshari et al. 2015; Pai and Ganesan 2018; Patel et al. 2022; Singh et al. 2021; Kotb et al. 2025), while one study (Kia et al. 2020) used systemic curcumin (nanocurcumin 80 mg capsule once daily for 4 weeks). The frequency of curcumin application varied across the included studies, ranging from 2 to 6 times per day. The concentration of curcumin was 1% in four studies (Thomas et al. 2017; Biswas et al. 2018; Patel et al. 2022; Kotb et al. 2025) and 5% in two studies (Kia et al. 2015; Tantia and Gupta 2021), while four studies (Nosratzehi et al. 2018; Keshari et al. 2015; Pai and Ganesan 2018; Singh et al. 2021) did not mention the concentration (Table 1).

With regard to the corticosteroid groups, 1% triamcinolone acetonide oral paste was used in nine studies (Kia et al. 2015; Tantia and Gupta 2021; Thomas et al. 2017; Biswas et al. 2018; Keshari et al. 2015; Pai and Ganesan 2018; Patel et al. 2022; Singh et al. 2021; Kotb et al. 2025), 1% betamethasone lotion in one study (Nosratzehi et al. 2018), and prednisone 10 mg tablets (systemically) in one study (Kia et al. 2020) (Table 1).

### 3.4 Outcomes Measures

With respect to pain/burning sensation, all included studies evaluated the efficacy of curcumin in alleviating pain/burning sensation, eight of which used Visual Analog Scale (Kia et al. 2020, 2015; Nosratzehi et al. 2018; Tantia and Gupta 2021; Biswas et al. 2018; Patel et al. 2022; Singh et al. 2021; Kotb et al. 2025), while three studies (Thomas et al. 2017; Keshari et al. 2015; Pai and Ganesan 2018) used Numerical Rating Scale.

Concerning the clinical improvement, all included studies reported on this outcome (at least one or more of the following parameters were evaluated in each included study: level of erythema; size of the ulcer/erosion) (Table 1).

### 3.5 Quality of the Included Studies

**Figure 2:**
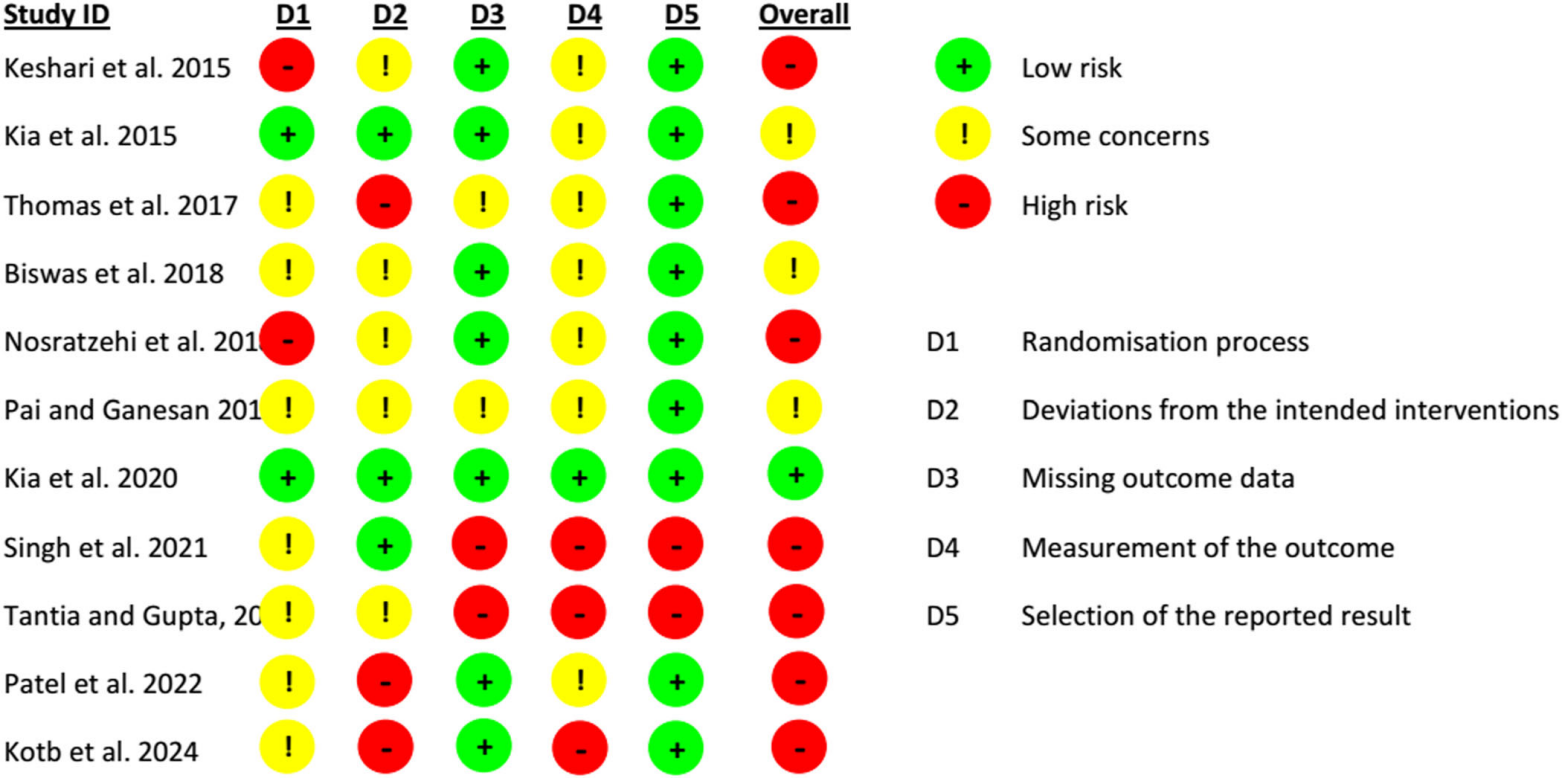
Risk of bias results.

Figure 2 illustrates the results of the ROB 2‐based quality of the included studies. Only one study (Kia et al. 2020) was at low ROB, while the remaining studies were either at high ROB (Nosratzehi et al. 2018; Tantia and Gupta 2021; Thomas et al. 2017; Keshari et al. 2015; Patel et al. 2022; Singh et al. 2021; Kotb et al. 2025) or showed “some concerns” (Kia et al. 2015; Biswas et al. 2018; Pai and Ganesan 2018) (Figure 2).

### 3.6 Qualitative Outcomes

All included studies found curcumin to be efficacious in reducing signs and symptoms of OLP. Seven studies (Kia et al. 2020, 2015; Nosratzehi et al. 2018; Tantia and Gupta 2021; Biswas et al. 2018; Patel et al. 2022; Singh et al. 2021) reported comparable efficacy of curcumin and corticosteroids in reducing pain and resolution of clinical signs without any significant differences between the two groups. Two studies (Keshari et al. 2015; Pai and Ganesan 2018) found curcumin to be more efficacious than corticosteroids in alleviating pain (Pai and Ganesan 2018) and erythema improvement (Keshari et al. 2015; Pai and Ganesan 2018). Conversely, one study (Thomas et al. 2017) reported superior results in favor of corticosteroids (Table 1).

### 3.7 Side Effects

Nine studies (Kia et al. 2020, 2015; Nosratzehi et al. 2018; Thomas et al. 2017; Biswas et al. 2018; Keshari et al. 2015; Pai and Ganesan 2018; Patel et al. 2022; Singh et al. 2021) reported on this outcome, while two studies (Tantia and Gupta 2021; Kotb et al. 2025) failed to mention any information in this regard. Four studies (Kia et al. 2015; Biswas et al. 2018; Keshari et al. 2015; Patel et al. 2022) reported side adverse effects in the corticosteroids group, mainly candidiasis and burning sensation. Except for one study (Kia et al. 2015), which revealed minimal adverse effects in some patients receiving curcumin that disappeared at the end of the first week of treatment, other studies reported no side effects in the curcumin group (Table 1).

### 3.8 Results Synthesis

**Figure 3:**
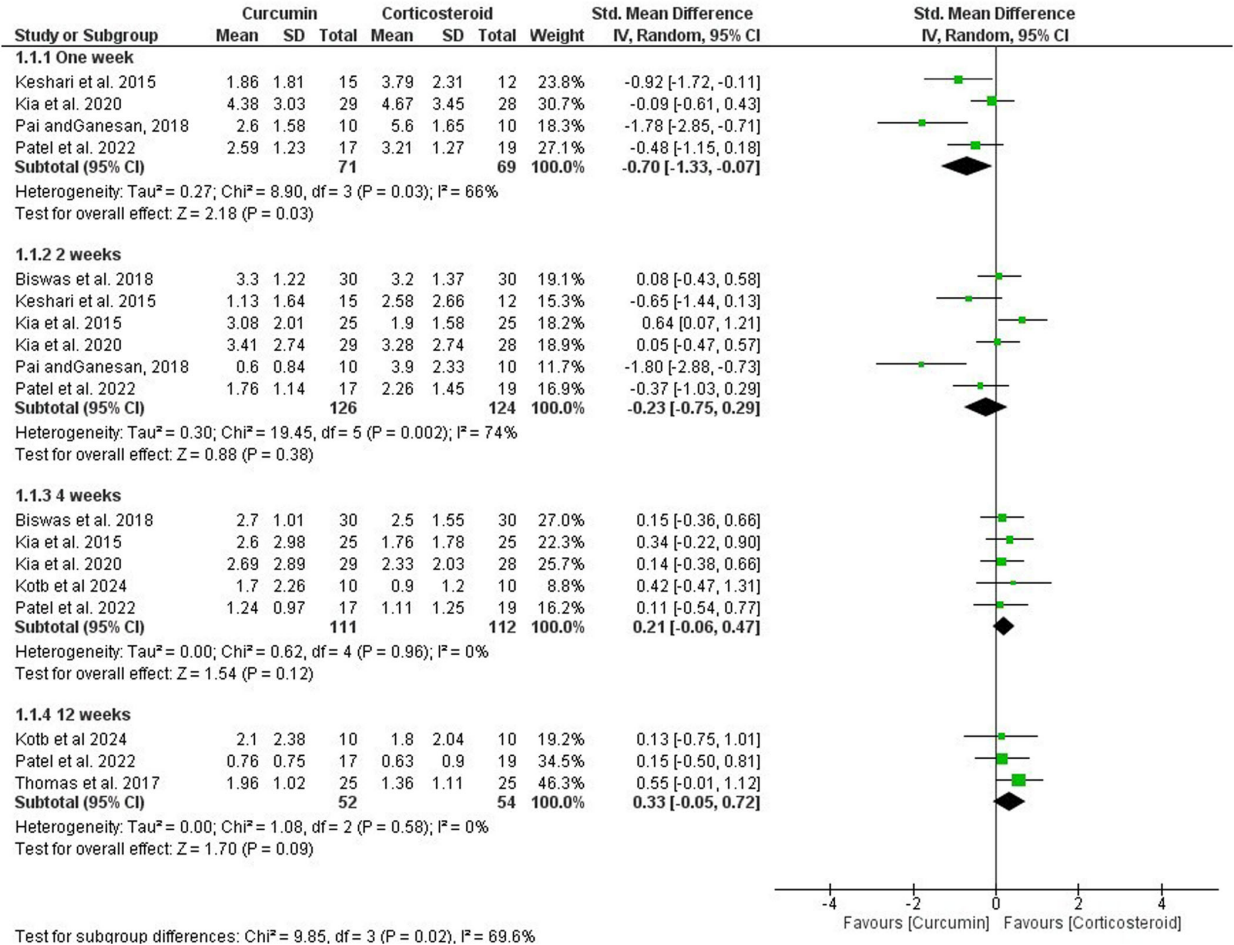
Forest plot of pain scores. CI, confidence interval; IV, instrumental variable.

**Figure 4:**
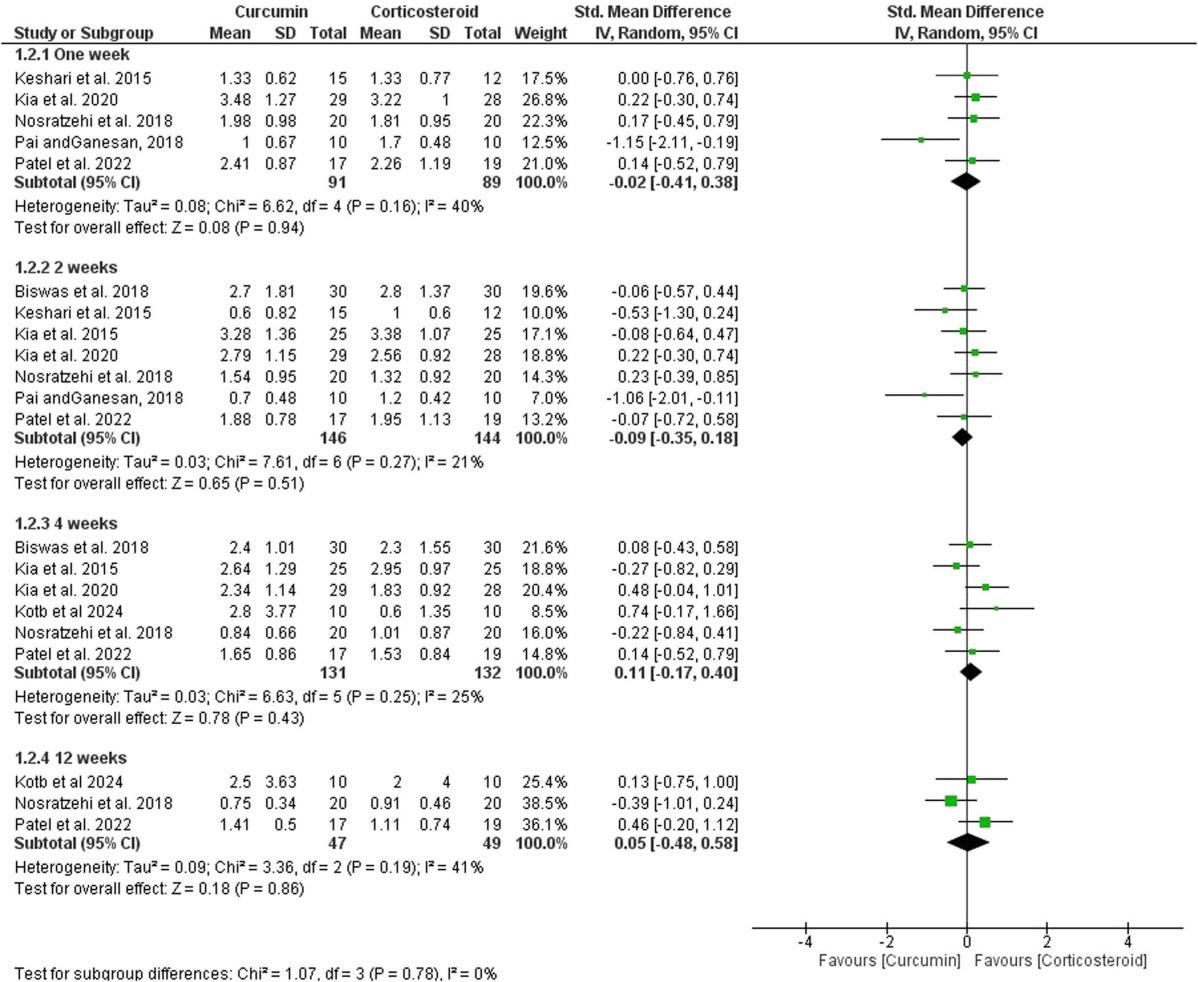
Forest plot of clinical improvement. CI, confidence interval; IV, instrumental variable.

The pooled data indicated a significantly greater efficacy of curcumin in alleviating pain at 1 week (SMD, −0.70; 95% CI, −1.33, −0.07; *p* = 0.03; *I* ^2^ = 66%) when compared with corticosteroids. However, comparable outcomes were observed at 2 weeks (SMD, −0.23; 95% CI, −0.75, 0.29; *p* = 0.38; *I* ^2^ = 74%), 4 weeks (SMD, 0.21; 95% CI, −0.06, 0.47; *p* = 0.96; *I* ^2^ = 0.0%), and 12 weeks (SMD, 0.33; 95% CI, −0.05, 0.82; *p* = 0.58; *I* ^2^ = 0.0%; Figure 3). Furthermore, the results revealed equivalent efficacy of curcumin and corticosteroids in achieving clinical improvement of the lesions across various time intervals, with no statistically significant differences detected between the two groups (*p* > 0.05; Figure 4).

#### 3.8.1 Publication Bias

The assessment of publication bias was not feasible due to the limited number of studies included; such analyses typically require a minimum of 10 studies to yield meaningful and reliable results.

## 4 Discussion

OLP is usually associated with severe pain and discomfort that adversely affects patients' quality of life (Carrozzo et al. 2019; Al‐Maweri, Al‐Jamaei, et al. 2018). Curcumin has emerged as a new alternative therapy for several oral inflammatory conditions, including OLP (Kia et al. 2015; Thomas et al. 2017; Al‐Maweri 2019; Al‐Maweri, Alaizari, et al. 2022; Al‐Maweri, Alhajj, et al. 2022; Dharman and Ravinthar 2020). In this regard, a number of clinical trials tested the efficacy of curcumin for the management of patients with OLP and revealed very promising results (Youssef et al. 2019; Hashem et al. 2019; Shetty et al. 2016; Nolan et al. 2009). Hence, the present systematic review and meta‐analysis investigated the available evidence regarding the clinical efficacy and safety of curcumin for the management of OLP. Overall, all included studies found curcumin to be efficacious in reducing the signs and symptoms of OLP. Additionally, the pooled data revealed that curcumin was more efficacious than corticosteroids in reducing pain at 1 week, but comparable with corticosteroids at 2‐, 4‐, and 12‐week follow‐up. Furthermore, the results revealed a comparable efficacy of curcumin and corticosteroids in clinical improvement of the lesions at different time intervals, with no statistically significant differences between the two groups (*p* > 0.05).

The key finding of the present review is the positive effects of curcumin in reducing pain associated with OLP. The immediate analgesic effects of curcumin are likely attributed to its strong anti‐inflammatory properties. Curcumin has been shown to have a potent anti‐inflammatory effect through inhibiting both the cyclooxygenase and lipoxygenase pathways of inflammation, thus inhibiting inflammatory mediators, such as prostaglandins and leukotrienes (Witkin and Li 2013; Ferguson et al. 2021; Uddin et al. 2021). Another important finding in the present study is the positive clinical efficacy of curcumin in improving the clinical signs of the diseases, as demonstrated by the significant reduction in the erythema and size of the ulcerative lesions. The positive clinical efficacy of curcumin can be explained by its immunomodulatory and antioxidant effects (Memarzia et al. 2021). It should be noted that although the exact etiopathogenesis of OLP is still obscure, mounting evidence indicates a T‐cell‐mediated immune disorder in response to the unknown trigger (DeAngelis et al. 2019). Curcumin has been shown to have a strong immunomodulatory effect through inhibiting T‐cell proliferation and suppressing cytokine production/expression (Yadav et al. 2005; Yuandani et al. 2021); this may explain its benefits in reducing signs and symptoms of OLP. Additionally, curcumin has strong antioxidant action through inhibiting the free radicals and reactive oxygen species (Memarzia et al. 2021), which may further explain the clinical efficacy of curcumin in OLP patients. By and large, it can be surmised that the synergistic anti‐inflammatory, immunomodulatory, and antioxidant properties of curcumin are responsible for its clinical benefits in OLP patients (Tang et al. 2021; Memarzia et al. 2021). These results support previous systematic reviews and meta‐analyses, which reported positive effects of curcumin in alleviating signs and symptoms associated with various oral mucosal lesions (Al‐Maweri 2019; Al‐Maweri, Alaizari, et al. 2022; Lv et al. 2019).

One important concern of OLP therapy is the unwanted side effects associated with conventional therapies, mainly corticosteroids. Of note, OLP is a chronic inflammatory disorder with recurrent remissions and exacerbations that necessitates long‐term use of topical and/or systemic corticosteroids either alone or in combination with other immunosuppressant agents (Lodi et al. 2020). Long‐term use of corticosteroids has been associated with several side effects, including candidiasis, xerostomia, and mucosal thinning (Su et al. 2022; Biswas et al. 2018). The current systematic review evaluated the safety of curcumin as a secondary outcome, and the results revealed that curcumin is safe and well‐tolerated with no or minimal side effects. In contrast, many patients in the corticosteroid groups showed several adverse effects, such as oral candidiasis and mucosal burning sensation. The results confirm the safety of curcumin and substantiate the previous evidence that reported similar results (Al‐Maweri 2019; Lv et al. 2019).

Indeed, the dose and formulation are important factors that influence the clinical efficacy of any medication, including curcumin. One limitation of curcumin is related to its poor bioavailability, mainly due to poor resorption (Sohn et al. 2021). In the present study, all included studies except one (Kia et al. 2020) used topical curcumin, with a frequency ranging from 2 to 6 times/day. One study by Thomas et al (Thomas et al. 2017). assessed the efficacy of two regimes of curcumin (one group received 1% curcumin gel three times/day and the other received 1% gel curcumin, six times/day) in comparison to topical corticosteroids, and concluded that the group receiving curcumin 6 times/day showed better efficacy than the one used it three times/day (Thomas et al. 2017). This confirms the importance of the drug's optimum dose for achieving good clinical efficacy. In the present study, one study compared systemic curcumin 80 mg capsule (nanocurcumin) with prednisolone 10 mg, and the results revealed very good efficacy of curcumin without any reported side effects. The authors concluded that systemic nanocurcumin is very efficacious and safe for the management of OLP. Hence, further clinical studies with standardized doses and formulations are required to discern the efficacy of curcumin and achieve the maximum clinical efficacy.

The present systematic review supports the efficacy of curcumin for the management of OLP. To the best of our knowledge, this is the first systematic review that summarizes the evidence regarding the efficacy of curcumin for OLP in comparison to corticosteroids. However, the present study has several limitations that should be noted. The primary limitation is the low quality of some of the included studies, as evidenced by the high ROB in eight of them, which prevents drawing firm conclusions. Another significant limitation is the considerable variability across the studies in terms of curcumin dosage and formulation, therapy duration, follow‐up period, OLP severity, and participant age and gender. Furthermore, in some studies, the diagnosis of OLP was based solely on clinical features, with no histopathological confirmation. Additionally, the majority of the studies were conducted in a single country (India), which may limit the generalizability of the results.

In short, the present systematic review and meta‐analysis reveal a good clinical efficacy of curcumin in alleviating the signs and symptoms of OLP. Further clinical trials with stringent methodologies and long follow‐up periods are highly recommended.

## Author Contributions

**Sadeq A. Al‐Maweri:** study conception, preparing the first draft, managing the project. **Gamilah Al‐Qadhi:** preparing the first draft, data curation, critically revising the manuscript. **Ahmed Yaseen Alqutaibi:** data analysis, critically revising the manuscript. **Nadhem M. Sallam:** study conception, data curation, critically revising the manuscript. **Mounzer Assad:** study, conception, data curation, critically revising the manuscript. **Mahfoudh A. Abdulghani:** preparing the first draft, data curation, critically revising the manuscript. **Marwan Mansoor Ali Mohammed:** study conception, data curation, critically revising the manuscript. All authors approved the final version of the manuscript.

## Conflicts of Interest

The authors declare no conflicts of interest.

## Supporting information


**Supplementary Table 1**: Databases: Applied search strategy, and numbers of retrieved studies. **Supplementary Table 2:** List of excluded studies and the reason of exclusion.

## Data Availability

The raw data of the present study are available from the corresponding author upon request.
