# Supplementary material for: Curcumin Versus Corticosteroids for Symptomatic Oral Lichen Planus: A Systematic Review and Meta‐Analysis
Source: Clin Exp Dent Res. 2025 Sep 22;11(5):e70227. doi: 10.1002/cre2.70227 (PMC12451056; doi:10.1002/cre2.70227)
Supplement: Supplementary file 1 — Supplementary Table 1: Databases: Applied search strategy, and numbers of retrieved studies. Supplementary Table 2: List of excluded studies and the reason of exclusion. [file CRE2-11-e70227-s001.docx]

| **Supplementary Table 1. Databases: Applied search strategy, and numbers of retrieved studies**  . | | | |
| --- | --- | --- | --- |
| Databases | Search strategy used | | Hits |
| MEDLINE searched via PubMed searched on  June 16, 2022, via www.ncbi .nlm.nih.gov/sites  Updated search on April 4, 2025 | | #1 Search ALL fields ("Lichen Planus, Oral"[Mesh] OR Oral lichen planus) 6040  #2 search ALL fields "Curcumin"[Mesh] OR curcumin OR turmeric  29, 293  #3 search #1 AND #2 32 | 32 |
| ISI web of science Core Collection was searched via web of knowledge on June 16, 2022, via: apps.webofknowledge.com  Updated search on April 4, 2025 | ((ALL=(Oral lichen planus )) AND ALL=(curcumin)) OR ALL=(turmeric ) | | 37 |
|  |  | |  |
| Scopus searched via Scopus on June 16, 2022, via <https://www.scopus.com>  Updated search on April 4, 2025 | ( TITLE-ABS-KEY ( oral AND lichen AND planus ) AND TITLE-ABS-KEY ( curcumin ) OR TITLE-ABS-KEY ( turmeric ) )  52 | | 77 |
| Google Scholar was searched via <https://scholar.google.com/> on June, 16, 2022  Updated search on April 4, 2025 | Oral lichen planus AND Curcumin OR turmeric | | 1800 |
|  | 1150 | |  |
| **Total** |  | | 1300 |

**Supplementary Table 2: List of excluded studies and the reason of exclusion**

| # | **Author, year** | **Title of the study** | **Reason for exclusion** |
| --- | --- | --- | --- |
| **1** | Chainani -Wu et al. 2007 | A randomized, placebo-controlled, double-blind clinical trial of curcuminoids in oral lichen planus. | Negative control (placebo) |
| **2** | Chainani -Wu et al. 2012 | High-dose curcuminoids are efficacious in the reduction in symptoms and signs of oral lichen planus. | Negative control (placebo) |
| **3** | Chainani -Wu et al. 2012 | Use of curcuminoids in a cohort of patients with oral lichen planus, an autoimmune disease. | No relevant control |
| **4** | Amirchaghmaghi et al. 2016 | Evaluation of the Efficacy of Curcumin in the Treatment of Oral Lichen Planus: A Randomized Controlled Trial. | Negative control (placebo) |
| **5** | Nigam et al. 2017 | Effect of Curcumin in Reducing Burning Sensation in Potentially Malignant Disorders of Oral Cavity. | No relevant control |
| **6** | Kapoor and Arora, 2019 | Effect of curcumin in management of potentially  Malignant disorders-A comparative study. | No relevant control |
| **7** | Bakhshi et al. 2020 | Combination Therapy with 1% Nanocurcumin Gel and 0.1% Triamcinolone Acetonide Mouth Rinse for Oral Lichen Planus: A Randomized Double-Blind Placebo Controlled Clinical Trial. | Combined therapy |
| **8** | Naik et al. 2021 | Curcumin alone and curcumin with prednisone in  management Oral Lichen Planus patients. | Combined therapy |
| **9** | Shivu et al. 2024 | Evaluation of Turmeric, Ashwagandha, and Aloe Vera in the Management of Oral Lichen Planus Lesion | No relevant control group |
| **10** | Khaitan 2022 | *Curcuma Longa* in the Treatment of Symptomatic oral lichen planus: A non-randomized controlled trial. | No relevant control group |
| **11** | GHOBADI et al 2022 | Effect of curcumin on oral lichen planus: A single blind randomized controlled clinical trial. | Placebo control group |

**References**

1. Chainani-Wu N, Silverman S, Jr., Reingold A, Bostrom A, Mc Culloch C, Lozada-Nur F, et al. A randomized, placebo-controlled, double-blind clinical trial of curcuminoids in oral lichen planus. Phytomedicine. 2007;14(7-8):437-46.

2. Chainani-Wu N, Madden E, Lozada-Nur F, Silverman S, Jr. High-dose curcuminoids are efficacious in the reduction in symptoms and signs of oral lichen planus. J Am Acad Dermatol. 2012;66(5):752-60.

3. Chainani-Wu N, Collins K, Silverman S, Jr. Use of curcuminoids in a cohort of patients with oral lichen planus, an autoimmune disease. Phytomedicine. 2012;19(5):418-23.

4. Amirchaghmaghi M, Pakfetrat A, Delavarian Z, Ghalavani H, Ghazi A. Evaluation of the Efficacy of Curcumin in the Treatment of Oral Lichen Planus: A Randomized Controlled Trial. J Clin Diagn Res. 2016;10(5):ZC134-7.

5. Nigam N, Chandra S, Reddy Enja S, Pandey N. Effect of curcumin in reducing burning sensation in potentially malignant disorders of oral cavity. Journal of Indian Academy of Oral Medicine and Radiology. 2017;29(1).

6. Kapoor S, Arora P. Effect of curcumin in management of potentially Malignant disorders-A comparative study. Oncology and Radiotherapy. 2019;1(46):4.

7. Bakhshi M, Gholami S, Mahboubi A, Jaafari MR, Namdari M. Combination Therapy with 1% Nanocurcumin Gel and 0.1% Triamcinolone Acetonide Mouth Rinse for Oral Lichen Planus: A Randomized Double-Blind Placebo Controlled Clinical Trial. Dermatol Res Pract. 2020;2020:4298193.

8. Naik D, Nazneen L, Dhobley A, Thombre A, Saxena U, Kosta S. Curcumin alone and curcumin with prednisone in management Oral Lichen Planuspatients. European Journal of Molecular & Clinical Medicine. 2021;8(3):5.

9. Shivu ME, Gupta N, Mulla M, Mulla M, Anand Bajoria A, Varshney A. Evaluation of Turmeric, Ashwagandha, and Aloe Vera in the Management of Oral Lichen Planus Lesion. J Pharm Bioallied Sci. 2024 Jul;16(Suppl 3):S2043-S2045. doi: 10.4103/jpbs.jpbs_1264_23. Epub 2024 May 1. PMID: 39346383; PMCID: PMC11426787.

10. Khaitan T, Vishal, Kabiraj A, Sinha DK, Ranjan R, Singh R. *Curcuma Longa* in the Treatment of Symptomatic oral lichen planus: A non-randomized controlled trial. Indian J Dermatol. 2022 Jul-Aug;67(4):478. doi: 10.4103/ijd.ijd_1065_20. PMID: 36578734; PMCID: PMC9792051.

11. GHOBADI, Nazanin; LESAN, Simin; KHATIBI, Mandana. Effect of curcumin on oral lichen planus: A single blind randomized controlled clinical trial. Journal of Mazandaran University of Medical Sciences, 2022, 32.211: 37-48.‏
